# Preliminary Analysis of the Nonsynonymous Polymorphism rs17563 in *BMP4* Gene in Brazilian Population Suggests Protection for Nonsyndromic Cleft Lip and Palate

**DOI:** 10.1155/2012/247104

**Published:** 2012-11-27

**Authors:** Tânia Kawasaki Araújo, Milena Simioni, Têmis Maria Félix, Liliane Todeschini de Souza, Marshall Ítalo Barros Fontes, Isabella Lopes Monlleó, Josiane Souza, Agnes Cristina Fett-Conte, Rodrigo Secolin, Iscia Lopes-Cendes, Cláudia Vianna Maurer-Morelli, Vera Lúcia Gil-da-Silva-Lopes

**Affiliations:** ^1^Department of Medical Genetics, Faculty of Medical Sciences, University of Campinas (UNICAMP), 13083-887 Campinas, SP, Brazil; ^2^Medical Genetics Service, Hospital de Clinicas de Porto Alegre, 90035-903 Porto Alegre, RS, Brazil; ^3^Medical Genetics Sector, State University of Alagoas, 57010-300 Maceio, AL, Brazil; ^4^Clinical Genetics Service, University Hospital, Federal University of Alagoas, 57072-970 Maceio, AL, Brazil; ^5^Centro de Atendimento Integral ao Fissurado Labiopalatal (CAIF), 81050-00 Curitiba, PR, Brazil; ^6^Department of Molecular Biology, Medicine School of São José do Rio Preto (FAMERP FUNFARME), 15090-000 São José do Rio Preto, SP, Brazil

## Abstract

Cleft lip with or without palate (CL±P) is common congenital anomalies in humans. Experimental evidence has demonstrated that bone morphogenetic protein 4 gene (*Bmp4*) is involved in the etiology of CL±P in animal models. The nonsynonymous polymorphism rs17563 T>C (p.V152A) in the *BMP4* gene has been associated to the risk of nonsyndromic CL±P in Chinese population and microforms from different ethnic backgrounds. The aim of this study was to investigate the role of *BMP4* gene in CL±P in Brazilian sample using genetic association approach. Our sample was composed by 123 patients with nonsyndromic CL±P and 246 controls, in which absence of CL±P was confirmed in 3 generations. The rs17563 polymorphism was genotyped by PCR-RFLP technique. Logistic regression was performed to evaluate allele and genotype association. Our data showed statistical power to detect association (86.83%) in this sample. Logistic regression results showed significant association between C allele and CL±P (*P* = 0.00018, OR = 0.40, and 95% CI = 0.25–0.65), as well as CC genotype and CL±P (*P* = 0.00018, OR = 0.35, and 95% CI = 0.19–0.66). So, there is a strong association between nonsyndromic CL±P and *BMP4* rs17563 polymorphism in our sample and the C allele had a protective effect against the occurrence of nonsyndromic CL±P.

## 1. Introduction 

Nonsyndromic cleft lip with or without palate (CL±P) is a congenital defect with multifatorial transmission. It is a birth defect that occurs with a prevalence of 1 in 500 to 1 in 2500 live births in different populations, varying with geographic location, ethnic group, and socioeconomic conditions [1–3]. Populations of either American native descent or Asian descent have the highest birth prevalence [4]. In Latin America, according to the ECLAMC (Latin American Collaborative Study of Congenital Malformations), the incidence of this malformation is 1 in 850 births [5]. CL±P has a complex etiology that includes strong genetic and environmental factors.

Clinically, CL±P are classified as nonsyndromic (isolated) or syndromic based on the presence of other congenital anomalies or mental disabilities [1, 6]. Around 400 recognizable syndromes that have CL±P as part of the phenotype are described [7, 8], however, 70% of CL±P cases are nonsyndromic. Approximately 12–25% of the genetic variations associated with nonsyndromic CL±P have been identified [1, 6]. Although genetic studies have identified a number of candidate genes and chromosomal regions associated with CL±P, findings from different studies have been inconsistent [9, 10].

Candidate genes for CL±P have been proposed as result of studies using knockout mice models. One interesting candidate gene is the Bone morphogenetic protein 4 (*BMP4*; MIM#112262). Experimental evidences have postulated that *Bmp4* is involved in the etiology of CL±P in mice. Bilateral cleft lip was presented in all knockout mice for *Bmp4* at 12 days after conception, however after 14.2 days only 22% of the embryos showed cleft lip. Moreover, Bmp signaling is also required for cell proliferation at earlier stages, in the maxillary process mesenchyme [11].

*BMP4* gene, located in 14q22-q23, is a member of the BMP family and a member of the transforming growth factor-b (TGF-b) of secretory signaling molecules that play essential roles in embryonic development [12]. This gene is a well-characterized mammalian growth factor. Expression of the *Bmp4* gene occurs in a wide spectrum of normal tissue in mouse embryos, as cardiac [11], limb [13], and face tissue [11, 14]. 

In addition, possible association between *BMP4* gene and nonsyndromic CL±P in humans was firstly suggested in a case-control study performed with Chinese children in 2008 [15]. Authors analyzed the single-nucleotide polymorphism (SNP) rs17563 (p.Val152Ala) and found an increased risk of nonsyndromic CL±P among carriers of the C allele.

A missense and nonsense mutations were found in *BMP4* gene in a sample of patients from different genetic background (Mongolia, Philippines, United States of America, Colombia, Guatemala, and Europe) diagnosed with subepithelial, microform, and overt cleft lip. These mutations were not found in a control population [16]. 

Furthermore, Suazo et al. [17] reported a mutation screening analysis of *BMP4* in a sample of 150 Chilean nonsyndromic CL±P case-parent trios. Significant deviations from expected transmissions were observed for haplotypes conformed by rs1957860 and rs762642. These polymorphisms delimitate a genomic region where a promoter and an enhancer of *BMP4* are located [18]. Then, Suazo et al. [19] searched for nonsyndromic CL±P risk variants within the two *BMP4* promoters by direct sequencing in a 167 Chilean nonsyndromic CL±P cases and 336 controls. They found three novel variants considered as cleft risk factors, as they are not present in controls.

Lately, Chen et al. [20] provided further evidence of association between *BMP4* gene and nonsyndromic CL±P. They tested for possible association between markers in and around the *BMP4* gene and nonsyndromic CL±P in Asian and Maryland trios. Nominal significant evidence of linkage and association was observed for three SNPs (rs10130587, rs2738265, and rs2761887) in 221 Asian trios and for one SNP (rs762642) in 76 Maryland trios. 

Brazilian population presents a heterogeneous ethnic origin which includes Native Americans, Europeans, Africans, and some degree of Asians. Relative contribution of each one of these ethnic groups is variable according to the geographic region of the country. This characteristic gives a unique and richly blended genetic and cultural background to the country [21–25].

Therefore, since Brazilian population presents a diverse genetic background, the purpose of this study was to investigate the role of the rs17563 polymorphism of *BMP4* gene in CL±P in a sample of Brazilian population.

## 2. Materials and Methods

### 2.1. Study Population

The sample consisted of 369 individuals enrolled in Craniofacial Brazil Project. All cases were evaluated by clinical geneticist using the same clinical protocol, including family history. Subjects were recruited between 2009 and 2011 from five centers of research involved in Craniofacial Brazil Project located in six cities from three different regions of Brazil (Southeast region: Campinas and São José do Rio Preto, South region: Porto Alegre and Curitiba, Northeast region: Maceió). Case group was compound of 123 individuals (62 males and 61 females) with nonsyndromic CL±P (98 cleft lip and palate and 25 cleft lip only). Control group were recruited from Southeast region by universitary e-mail included 246 phenotypically normal unrelated individuals (102 males and 144 females) without family history of oral cleft in three generations and no Japanese or Chinese ancestry. Their family history was collected by a participant researcher.

The study was approved by the local Institutional Review Boards and the National Research Ethics Committee (CEP 059/2008). All participants provided informed consent. The informed consent for patients under 18 years old was obtained from their parents. 

### 2.2. Molecular Analysis

Genomic DNA was extracted from peripheral blood lymphocytes according to the method described by De Araujo et al. [26].

SNP genotyping was performed using a polymerase chain reaction-restriction fragment length polymorphism (PCR-RFLP) assay. The PCR primers were designed based on the SNP flanking sequence described in the Ensembl Genome Browser (http://www.ensembl.org/index.html). 

Primer sequences for PCR reactions were 5′AGTTTGGCTGCTTCTCCC3′ (forward) and 5′AGTTTGGCTGCTTCTCCC3′ (reverse). The PCR reaction were performed in a total volume of 15 *μ*L containing 1.5 *μ*L KCl_2_ (10X), 0.6 *μ*L MgCl_2_ (25 *μ*M), 1.5 *μ*L dNTP (2 *μ*M), 0.35 *μ*L of each primer* primer sense* (5pmoles), 0.5 *μ*L DMSO_4_, 0.2 *μ*L Taq DNA Polimerase (5U) (New England BioLabs, Ipswich, MA, USA), and 1 *μ*L DNA (200 ng/*μ*L) e 9 *μ*L H_2_O. The PCR cycle conditions consisted of an initial denaturation step at 94°C for 5 min followed by 35 cycles of 1 min at 94°C, 1 min at 56°C, 1 min at 72°C, and a final elongation at 72°C for 7 min.

Adequate restriction endonuclease for SNP was selected using the Gene Runner software (Hastings Software Inc., Hastings, NY, USA; http://www.generunner.net/). Primers, restriction enzyme, and the length of digested fragments for the SNP are listed in Table 1. 

The digestion reaction were performed in a total volume of 20 *μ*L containing 15 *μ*L PCR, 0.5 *μ*L enzyme, and 2.0 *μ*L buffer e 2.5 *μ*L H_2_O. The PCR products were digested overnight at 37°C with *Hph*I enzyme (New England BioLabs, Ipswich, MA, USA). PCR products were electrophoresed through 1% agarose gel in the presence of ethidium bromide and visualized by fluorescence in UV light; the digested PCR products were resolved on 12% polyacrylamide gels and visualized by a silver staining protocol.

### 2.3. SNP Association Analysis

Gender distribution was evaluated using Fisher's exact test in *R* statistical environment [27]. Minor allele frequency (MAF) and Hardy-Weinberg equilibrium were estimated using Haploview software [28] (http://www.broadinstitute.org/haploview). Allele and genotype association analysis were performed by logistic regression using *R* statistical environment [27]. We evaluated posthoc statistical power of our sample by GPOWER software [29], using the following parameters: logistic regression test; two-tail; OR = 1.5; statistical significance level *α* = 0.05; *n* = 369.

### 2.4. **In Silico ** Test

*In silico* tests were performed to analyze whether the substitution c.538T>C in the *BMP4* gene (rs17563) modifies the structure or function of the encoded protein. The sequence of amino acids used in the tests was obtained from NCBI database (http://www.ncbi.nlm.nih.gov/).

Three *in silico *tests were used: Grantham scale [30], PolyPhen [31] (http://genetics.bwh.harvard.edu/pph/), and SNAP [32] (http://rostlab.org/services/snap/) for the analysis of rs17563.

## 3. Results

The gender distribution was similar between groups (Fisher's exact test *P* = 0.1198). MAF was 0.478 and SNP rs17563 was in Hardy-Weinberg equilibrium (*P* = 0.9664) in the sample. There were significant differences in the genotypes and allele frequencies of the rs17563 between nonsyndromic CL±P and control groups.

Logistic regression revealed association between C allele and CL±P (*P* = 0.00018, OR = 0.40, and 95% CI = 0.25–0.65). Genotype logistic regression analysis showed rs17563 CC genotype was associated with decreased of CL±P susceptibility (*P* = 0.00081, OR = 0.35, and 95% CI = 0.19–0.66) (Table 2). The sample analyzed showed statistical power to detect genetic association (86.83%).

The results obtained of *in silico* tests were similar for the three. Grantham's scale showed that SNP was considered “moderately conservative” (value 64). The PolyPhen algorithm aligned sequences “wild” and “altered” to 75 homologous sequences and estimated the change as “benign” (score 0416). In addition, SNAP demonstrated that p.Val152Ala is neutral (reliability index = 3, expected accuracy = 78%).

## 4. Discussion

Mutant mice for *Bmp4* gene with CL±P phenotype suggest a possible role for this gene in lip and palate development [11]. Moreover, have been reported few studies of association between *BMP4* gene and CL±P in population. 

Lin et al. [15] found significant differences in genotype distribution and allele polymorphism rs17563 of *BMP4 *gene between 184 nonsyndromic CL±P cases and 205 controls. In a study with 1614 individuals from different countries (Mongolia, Philippines, United States of America, Colombia, Guatemala, and Europe), missense and nonsense mutations in *BMP4* gene were detected in 5 of 968 individuals with overt CL±P, 1 of 30 cases with microforms and 2 of 87 cases with orbicular oris muscle. They also found a borderline difference in the frequency of SNP rs17563 in cases compared with controls [16]. Thus, the results of this study support the hypothesis that this polymorphism might be clinically important in the genesis of CL±P.

Recently, Chen et al. [20] tested for possible association between markers in* BMP4* gene and nonsyndromic CL±P in 297 Asian and Maryland trios. Their results does not support the evidence of linkage and association for the SNP rs17563. 

However, in the present study the polymorphism rs17563 in *BMP4* gene was associated with decreased susceptibility for nonsyndromic CL±P (*P* = 0.00081, OR = 0.35, and 95% CI = 0.19–0.66). This result was the opposite found by Lin et al. [15] and Suzuki et al. [16]. The difference could be attributed to the clinical selection of individuals, the well-defined control group, and the statistics power herein used. As well, this study obtained a strong association between nonsyndromic CL±P and *BMP4* rs17563 polymorphism and suggested that C allele had a protective role in the occurrence of nonsyndromic CL±P in the Brazilian sample. 

The rs17563 polymorphism replaces the amino acid valine by alanine at position 152 of the protein. It would seem that the structure and function of the protein are not significantly affected by the substitution due to physicochemical similarities between the involved amino acids. In our study, results of the *in silico* test using Grantham's scale, PolyPhen, and SNAP were similar and showed the moderately conservative, benign, and neutral character of the rs17563 polymorphism. These results corroborate the hypothesis of a protective effect of this polymorphism. 

Although cleft lip with cleft palate and cleft lip only are usually grouped into the same category, Dixon et al. [33] suggested that they could be etiologically distinct. Thus, further genetic association studies designed to examine these two conditions separately would be helpful in elucidating the role *BMP4* rs17563 in orofacial clefts. 

## 5. Conclusions

This study found a strong association between nonsyndromic CL±P and *BMP4* rs17563 polymorphism and a possible protective effect of the C allele against nonsyndromic CL±P in a Brazilian sample. In this regard it could be speculated that rs17563 polymorphism plays different roles in oral cleft pathogenesis in specific populations. It would be interesting to replicate this study in individuals with similar and different ethnicity. As well, this association reinforces the importance of this gene as candidate for oral cleft.

## Figures and Tables

**Table 1:** Primers, restriction enzyme, and fragment lengths for rs17563 of *BMP4* gene.

SNP	Primer	Annealing temperature	Restriction enzyme	Fragment lengths
rs17563	Forward 5′CACCATTCATTGCCCAAC3′	56°C	HphI	T Allele: 195pb + 229pb
	Reverse 5′AGTTTGGCTGCTTCTCCC3′			C Allele: 424pb

**Table 2:** The genotype and allele distribution of rs17563 in nonsyndromic CL±P patients and controls and result of logistic regression.

SNP rs17563		Controls *n* (%)	Cases *n* (%)	*χ* ^2^	*P*	OR	95% CI
Genotype	TT^1^	52 (21,1)	49 (39,8)	—	—	1.00	—
	TC	130 (52,8)	53 (43,1)	10.645	0.00110	0.43	0.26–0.71
	CC	64 (26,1)	21 (17,1)	11.217	0.00081	0.35	0.19–0.66

Allele	T^1^	234 (47,6)	151 (61,4)	—	—	1.00	—
	C	258 (52,4)	95 (38,6)	13.992	0.00018	0.40	0.25–0.65

^1^Reference factor.

## References

[1] Schutte BC, Murray JC (1999). The many faces and factors of orofacial clefts. *Human Molecular Genetics*.

[2] Bender PL (2000). Genetics of cleft lip and palate. *Journal of Pediatric Nursing*.

[3] Murray JC (2002). Gene/environment causes of cleft lip and/or palate. *Clinical Genetics*.

[4] Forrester MB, Merz RD (2004). Descriptive epidemiology of oral clefts in a multiethnic population, Hawaii, 1986–2000. *Cleft Palate-Craniofacial Journal*.

[5] ECLAMC (Latin American Collaborative Study of Congenital Malformations) final document. Angra dos Reis: ECLAMC VI.

[6] Cobourne MT (2004). The complex genetics of cleft lip and palate. *European Journal of Orthodontics*.

[7] Lidral AC, Moreno LM (2005). Progress toward discerning the genetics of cleft lip. *Current Opinion in Pediatrics*.

[8] Sivertsen Å, Wilcox AJ, Skjærven R (2008). Familial risk of oral clefts by morphological type and severity: population based cohort study of first degree relatives. * British Medical Journal*.

[9] Vieira AR, Avila JR, Daack-Hirsch S (2005). Medical sequencing of candidate genes for nonsyndromic cleft lip and palate.. *PLoS Genetics*.

[10] Mostowska A, Hozyasz KK, Wojcicki P, Biedziak B, Paradowska P, Jagodzinski PP (2010). Association between genetic variants of reported candidate genes or regions and risk of cleft lip with or without cleft palate in the polish population. *Birth Defects Research A*.

[11] Liu W, Sun X, Braut A (2005). Distinct functions for Bmp signaling in lip and palate fusion in mice. *Development*.

[12] Leong LM, Brickell PM (1996). Bone morphogenetic protein-4. *International Journal of Biochemistry and Cell Biology*.

[13] Bandyopadhyay A, Tsuji K, Cox K, Harfe BD, Rosen V, Tabin CJ (2006). Genetic analysis of the roles of *BMP2*, *BMP4*, and *BMP7* in limb patterning and skeletogenesis.. *PLoS Genetics*.

[14] Gong SG, Guo C (2003). *Bmp4* gene is expressed at the putative site of fusion in the midfacial region. *Differentiation*.

[15] Lin JY, Chen YJ, Huang YL (2008). Association of bone morphogenetic protein 4 gene polymorphisms with nonsyndromic cleft lip with or without cleft palate in Chinese children. *DNA and Cell Biology*.

[16] Suzuki S, Marazita ML, Cooper ME (2009). Mutations in *BMP4* are associated with Subepithelial, Microform, and Overt Cleft Lip. *American Journal of Human Genetics*.

[17] Suazo J, Santos JL, Jara L, Blanco R (2010). Association between bone morphogenetic protein 4 gene polymorphisms with nonsyndromic cleft lip with or without cleft palate in a chilean population. *DNA and Cell Biology*.

[18] Kawai S, Sugiura T (2001). Characterization of human bone morphogenetic protein (BMP)-4 and -7 gene promoters: activation of BMP promoters by Gli, a sonic hedgehog mediator. *Bone*.

[19] Suazo J, Tapia JC, Santos JL, Castro VG, Colombo A, Blanco R (2011). Risk variants in *BMP4* promoters for nonsyndromic cleft lip/palate in a Chilean population. *BMC Medical Genetics*.

[20] Chen Q, Wang H, Hetmanski JB (2012). *BMP4* was associated with NSCL/P in an Asian population. *PLOS ONE*.

[21] Pellegrino J, Castilho L, Rios M, De Souza CA (2001). Blood group genotyping in a population of highly diverse ancestry. *Journal of Clinical Laboratory Analysis*.

[22] Callegari-Jacques SM, Grattapaglia D, Salzano FM (2003). Historical genetics: spatiotemporal analysis of the formation of the Brazilian population. *American Journal of Human Biology*.

[23] Ferreira LB, Mendes-Junior CT, Wiezel CEV, Luizon MR, Simões AL (2006). Genomic ancestry of a sample population from the State of São Paulo, Brazil. *American Journal of Human Biology*.

[24] Luizon MR, Mendes CT, De Oliveira SF, Simões AL (2008). Ancestry informative markers in Amerindians from Brazilian Amazon. *American Journal of Human Biology*.

[25] Muniz YCN, Ferreira LB, Mendes-Junior CT, Wiezel CEV, Simões AL (2008). Genomic ancestry in urban Afro-Brazilians. *Annals of Human Biology*.

[26] De-Araujo M, Sanches MR, Suzuki LA, Guerra G, Farah SB, De-Mello MP (1996). Molecular analysis of *CYP21* and *C4* genes in Brazilian families with the classical form of steroid 21-hydroxylase deficiency. *Brazilian Journal of Medical and Biological Research*.

[27] R Development Core Team (2010). *R: A Language and Environment For Statistical Computing*.

[28] Barrett JC, Fry B, Maller J, Daly MJ (2005). Haploview: analysis and visualization of LD and haplotype maps. *Bioinformatics*.

[29] Faul F, Erdfelder E, Lang AG, Buchner A (2007). G∗Power 3: a flexible statistical power analysis program for the social, behavioral, and biomedical sciences. *Behavior Research Methods*.

[30] Grantham R (1974). Amino acid difference formula to help explain protein evolution. *Science*.

[31] Ramensky V, Bork P, Sunyaev S (2002). Human non-synonymous SNPs: server and survey. *Nucleic Acids Research*.

[32] Thusberg J, Olatubosun A, Vihinen M (2011). Performance of mutation pathogenicity prediction methods on missense variants. *Human Mutation*.

[33] Dixon MJ, Marazita ML, Beaty TH, Murray JC (2011). Cleft lip and palate: understanding genetic and environmental influences. *Nature Reviews Genetics*.

